# *Aspilota isfahanensis*, a new species of the genus *Aspilota* Foerster, 1863 from Iran (Hymenoptera, Braconidae, Alysiinae)

**DOI:** 10.3897/zookeys.582.7426

**Published:** 2016-04-21

**Authors:** Francisco Javier Peris-Felipo, Zahra Yari, Ehsan Rakhshani, Sergey A. Belokobylskij

**Affiliations:** 1 Bleichestrasse 15, CH–4058 Basel, Switzerland; 2 Department of Plant Protection, College of Agriculture, University of Zabol, Zabol, P. O. Box: 98615-538, I. R. Iran; 3 Zoological Institute Russian Academy of Sciences, St Petersburg, 199034, Russia; Museum and Institute of Zoology Polish Academy of Sciences, Wilcza 64, Warszawa 00–679, Poland

**Keywords:** Alysiinae, Aspilota, new species, identification key, Palaearctic, Iran

## Abstract

A new species of *Aspilota* without mesoscutal pit, *Aspilota isfahanensis* Peris-Felipo, **sp. n**., is described and illustrated from Iran. The new species is compared with its three morphologically most similar species, *Aspilota compressiventris* Stelfox & Grahan, 1951, *Aspilota makita* Papp, 2008 and *Aspilota spiracula* Munk & Peris-Felipo, 2013, is provided. A key to the western Asian species of *Aspilota* is provided.

## Introduction

The complex of genera that are closely related to *Aspilota* is the most taxonomically complicated group within the braconid Alysiinae, mainly because of their small body size and their reduced number of available diagnostic characters (Belokobylskij 2005). The genus *Aspilota* Foerster, 1863, is well defined by the presence of the paraclypeal fovea connecting with inner margin of eye and of the vein cuqu1 (2-SR) of the fore wing (van Achterberg 1988; Peris-Felipo and Belokobylskij 2016).

Information about *Aspilota* species from Western Asia is scarce, and only two species have been previously recorded, both from Iran (Yu et al. 2012; Gadallah et al. 2015). In this work, an additional new species of *Aspilota* from Iran (Isfahan Province) is described. The new species is compared with its three morphologically similar Palaearctic species, *Aspilota compressiventris* Stelfox & Grahan, 1951, *Aspilota makita* Papp, 2008 and *Aspilota spiracula* Munk & Peris-Felipo, 2013, is provided. Finally, a key to the three western Asian species of *Aspilota* is given.

## Material and methods

For the terminology of the morphological features, sculpture and measurements, see Peris-Felipo et al. (2014); for wing venation nomenclature, see Peris-Felipo et al. (2014) and in parenthesis van Achterberg (1993). The keys by Fischer (1976, 1978), Belokobylskij and Tobias (2007) and Papp (2008) were used for the identification of the new *Aspilota* species. The material was imaged using Digital Microscope Keyence® VHX-2000 and Adobe Photoshop® imaging system. The types of the new species are deposited in the collections of the Naturhistorisches Museum (Vienna, Austria; NHMW) and Zoological Institute of the Russian Academy of Sciences (St Petersburg, Russia; ZISP).

## Taxonomy

### Order Hymenoptera L., 1758 Family Braconidae Nees, 1811 Subfamily Alysiinae Leach, 1815 Genus *Aspilota* Foerster, 1863

#### Aspilota isfahanensis

Taxon classificationAnimaliaHymenopteraBraconidae

Peris-Felipo sp. n.

http://zoobank.org/A4282F26-0353-4FFC-B8B5-2A13784E3C2B

Figs 1 , 2

##### Type material.

Holotype: female, Iran, Isfahan, 6.x.2012, sweep net on *Chenopodium* sp. (E. Nader leg.) (NHMW). Paratype: 1♀, same data as for holotype (ZISP).

##### Description.

Female (holotype).

**Figure 1.**
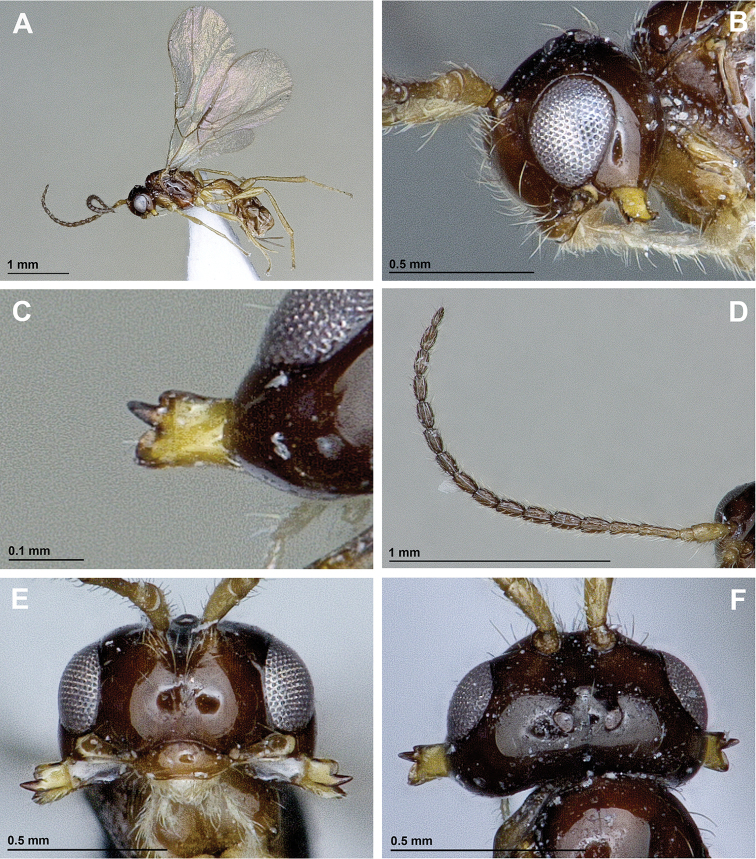
*Aspilota isfahanensis* sp. n. (female, holotype). **A** Habitus, lateral view **B** Head, lateral view **C** Mandible **D** Antenna **E** Head, front view **F** Head, dorsal view.

**Figure 2.**
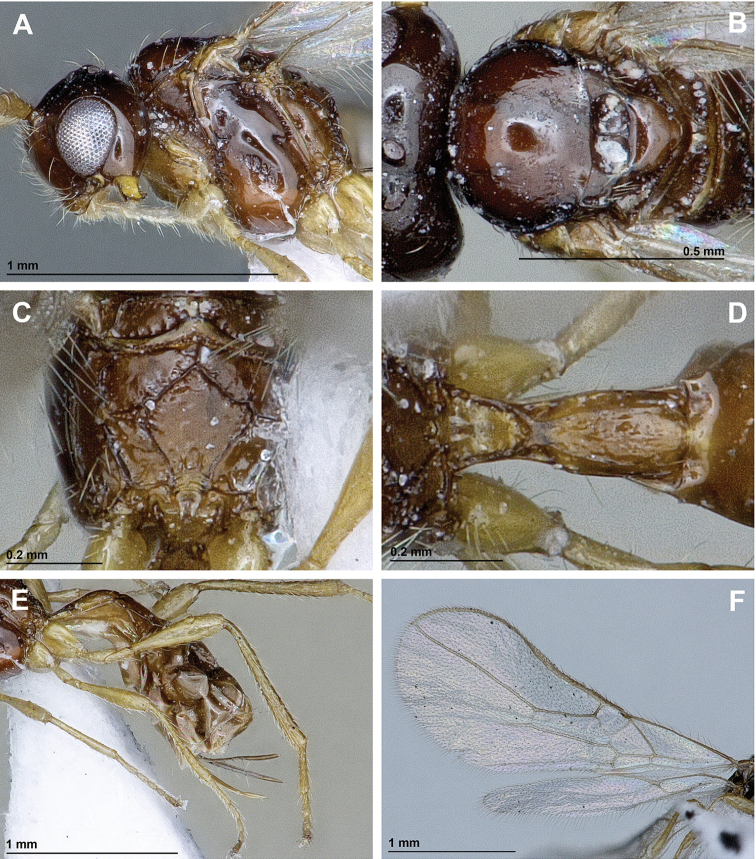
*Aspilota isfahanensis* sp. n. (female, holotype). **A** Head and mesosoma, lateral view **B** Mesonotum **C** Propodeum **D** First metasomal tergite **E** Hind leg, metasoma and ovipositor, lateral view **F** Fore and hind wings.

*Head*. In dorsal view, 1.9 times as wide as its median long, 1.4 times as wide as mesoscutum, smooth, with temple rounded behind eyes (Fig. 1F). Eye in lateral view 1.4 times as high as wide and 1.7 times as wide as temple medially (Figs 1B, 2A). POL 1.6 times OD; OOL 3.0 times OD (Fig. 1F). Face 1.9 times as wide as high; inner margins of eyes subparallel (Fig. 1E). Clypeus 2.5 times as wide as high, slightly curved ventrally (Fig. 1E). Paraclypeal fovea reaching inner margin of eye (Fig. 1E). Mandible 3-dentate, weakly widened towards apex, 1.3 times as long as its maximum width. Upper tooth distinctly shorter then lower tooth, very small and rounded; middle tooth rather long and narrow, longer than lower tooth, pointed apically; lower tooth widest, rounded, distinctly moving downwards (Fig. 1C). Antennae 17-segmented, 0.8 times as long as body. Scape 2.4 times longer than pedicel. First flagellar segment 3.3 times as long as its apical width, 1.3 times as long as second segment. Second flagellar segment 2.2 times as long as its maximum width; third to twelfth segments about 1.8 times as long as their maximum width, 13th and 14th segments 2.0 times, and 15th (apical) segment 2.5 times as long as their wide accordingly (Fig. 1D).

*Mesosoma*. In lateral view, 1.2 times as long as high (Fig. 2A). Mesoscutum (dorsal view) 0.8 times as long as its maximum width, smooth, with two lines of sparse setae along tracks of notauli (Fig. 2B). Notauli mainly absent on horizontal surface of mesoscutum (Fig. 2B). Mesoscutal pit absent (Fig. 2B). Prescutellar depression smooth, without lateral carinae (Fig. 2B). Precoxal sulcus present, crenulate, not reaching anterior and posterior margins of mesopleuron (Fig. 2A). Posterior mesopleural furrow crenulate in upper half, smooth in lower half (Fig. 2A). Propodeum with pentagonal areola delineated by distinct carinae (Fig. 2C). Propodeal spiracles relatively small (Fig. 2C).

*Wings* (Fig. 2F). Length of fore wing 2.7 times as long as its maximum width. Radial (marginal) cell ending at apex of wing, 4.0 times as long as its maximum width. Vein r2 (3-SR) 2.3 times as long as vein cuqu1 (2-SR); vein r3 (SR1) 2.5 times as long as vein r2 (3-SR). Nervulus (cu-a) distinctly postfurcal. Brachial (subdiscal) cell closed distally, 3.0 times as long as its maximum width. Hind wing 6.5 times as long as its maximum width.

*Legs* (Fig. 2E). Hind femur claviform, 4.0 times as long as its maximum width. Hind tibia weakly widened towards apex, 9.7 times as long as its maximum subapical width, 1.5 times as long as its hind tarsus. First segment of hind tarsus twice as long as second segment.

*Metasoma*. First tergite long, slightly widened towards apex, 2.6 times as long as its apical width, finely rugose-striate in apical half (Fig. 2D). Ovipositor 1.2 times as long as first tergite, 0.4 times as long as metasoma, 0.9 times as long as hind femur, 0.2 times as long as fore wing (Fig. 2E).

*Colour*. Body, antenna, and pterostigma dark brown. Mandibles and legs yellowish brown. Wings hyaline. *Length*. Body 1.8 mm; fore wing 2.0 mm; hind wing 1.8 mm. *Variation*. Antenna 16–17-segmented.

Male. Unknown.

##### Etymology.

Named after Isfahan, the type locality of new species.

##### Comparative diagnosis.

This new species is similar to *Aspilota compressiventris* Stelfox & Grahan, 1951 (Austria, Hungary, Russia, and U.K), *Aspilota makita* Papp, 2008 (Hungary and Romania) and *Aspilota spiracula* Munk & Peris-Felipo, 2013 (Denmark). All these species have the propodeum with a pentagonal areola delineated by a distinct carinae. However, *Aspilota isfahanensis* sp. n. differs from *Aspilota compressiventris* in having the mandible 1.3 times as long as its maximum width (1.7 times in *Aspilota compressiventris*), the first flagellar segment 3.3 times as long as its maximum width (4.0 times in *Aspilota compressiventris*), the hind femur 4.0 times as long as its maximum width (4.5 times in *Aspilota compressiventris*), the first metasomal tergite 2.6 times as long as its apical width (3.0–4.0 times in *Aspilota compressiventris*), the face 1.9 times as long as high (1.5 times in *Aspilota compressiventris*), and the head in dorsal view 1.9 times as long as long (1.5 times in *Aspilota compressiventris*). The new species differs from *Aspilota makita* in having the mandible 1.3 times as long as its maximum width (1.7 times in *Aspilota makita*), a hind femur 4.0 times as long as its maximum apical (3.2 times in *Aspilota makita*), the first metasomal tergite 2.6 times as long as its apical width (2.0 times in *Aspilota makita*), a propodeum with the areola distinctly delineated by carinae (areola less distinctly delineated in *Aspilota makita*), the first flagellar segment 3.3 times as long as its maximum width (4.0 times in *Aspilota makita*), and the upper tooth rounded (pointed in *Aspilota makita*). Finally, *Aspilota isfahanensis* sp. n. differs from *Aspilota spiracula* in having the mandible 1.3 times as long as its maximum width (1.5 times in *Aspilota spiracula*), the eye in lateral view 1.7 times as wide as the temple medially (nearly as long in *Aspilota spiracula*), the first flagellar segment 3.3 times as long as its maximum width (2.5 times in *Aspilota spiracula*), middle flagellar segments 1.8–2.2 times as long as their maximum widths (1.0–1.1 times *Aspilota spiracula*), the first metasomal tergite 2.6 times as long as its apical width (2.3 times in *Aspilota spiracula*), and a long metasoma (short in *Aspilota spiracula*).

### Key to the western Asian species of *Aspilota*

**Table:** 

1	Eye in lateral view 0.5–0.8 times as wide as temple medially. First metasomal tergite about 2.0 times as long as its apical width. Hind femur 4.5–5.0 times as long as its maximum width	**2**
–	Eye in lateral view 1.7 times as wide as temple medially (Fig. 1B, 2A). First metasomal tergite 2.6 times as long as its apical width (Fig. 2D). Hind femur 4.0 times as long as its maximum width (Fig. 2E). Body length 1.8 mm. Iran	***Aspilota isfahanensis* Peris-Felipo, sp. n.**
2	Eye in lateral view 0.5 times as wide as temple medially. First flagellar segment 4.0 times as long as its maximum width; middle segments 1.5 times as long as their maximum width. Hind femur 5.0 times as long as its maximum width. Vein r2 (3-SR) 2.0 times as long as vein cuqu1 (2-SR). Body length 1.8 mm. Iran	***Aspilota alfalfae* Fischer, Lashkari Bod, Rakhshani & Talebi, 2011**
–	Eye in lateral view 0.8 times as wide as temple medially. First flagellar segment 3.5 times as long as its maximum width; middle segments 1.8 times as long as their maximum width. Hind femur 4.5 times as long as its maximum width. Vein r2 (3-SR) 2.5 times as long as vein cuqu1 (2-SR). Body length 1.8–2.2 mm. Austria, Greece, Hungary, Iran	***Aspilota delicata* Fischer, 1973**

## Supplementary Material

XML Treatment for Aspilota isfahanensis
